# Distúrbio Cumulativo da Lipofuscina Miocárdica após Transplante Cardíaco de Longa Evolução: Estudo Baseado em Biópsias Endomiocárdicas

**DOI:** 10.36660/abc.20220313

**Published:** 2023-03-27

**Affiliations:** 1 Universidade de São Paulo Faculdade de Medicina Hospital das Clínicas São Paulo SP Brasil Instituto do Coração do Hospital das Clínicas da Faculdade de Medicina da Universidade de São Paulo, São Paulo, SP – Brasil

**Keywords:** Transplante de Coração, Lipofuscina, Biópsia Endomiocárdica

## Introdução

Lipofuscina é um pigmento citoplasmático castanho-amarelado, não degradável, composto por proteínas altamente oxidadas, lípides e metais. A lipofuscina se acumula com o tempo em células pós-mitóticas perenes, como os cardiomiócitos, sendo também chamada “pigmento da idade”.^1-4^

O transplante cardíaco é tratamento consagrado para pacientes com insuficiência cardíaca grave refratária a outras formas de tratamento.^5^ Sua maior limitação no longo prazo é o desenvolvimento da doença vascular do enxerto (DVE), de diagnóstico relativamente difícil e para a qual há poucas opções efetivas de tratamento.^6,7^ Nesse contexto é de se salientar estudo prévio relacionando a quantidade de lipofuscina no miocárdio de pacientes transplantados cardíacos ao desenvolvimento da DVE.^8^

O objetivo deste trabalho foi avaliar a deposição de lipofuscina no miocárdio após o transplante cardíaco de longa evolução, e investigar a relação entre a quantidade de lipofuscina no miocárdio e a presença e gravidade da DVE.

## Métodos

**Figura 1:**
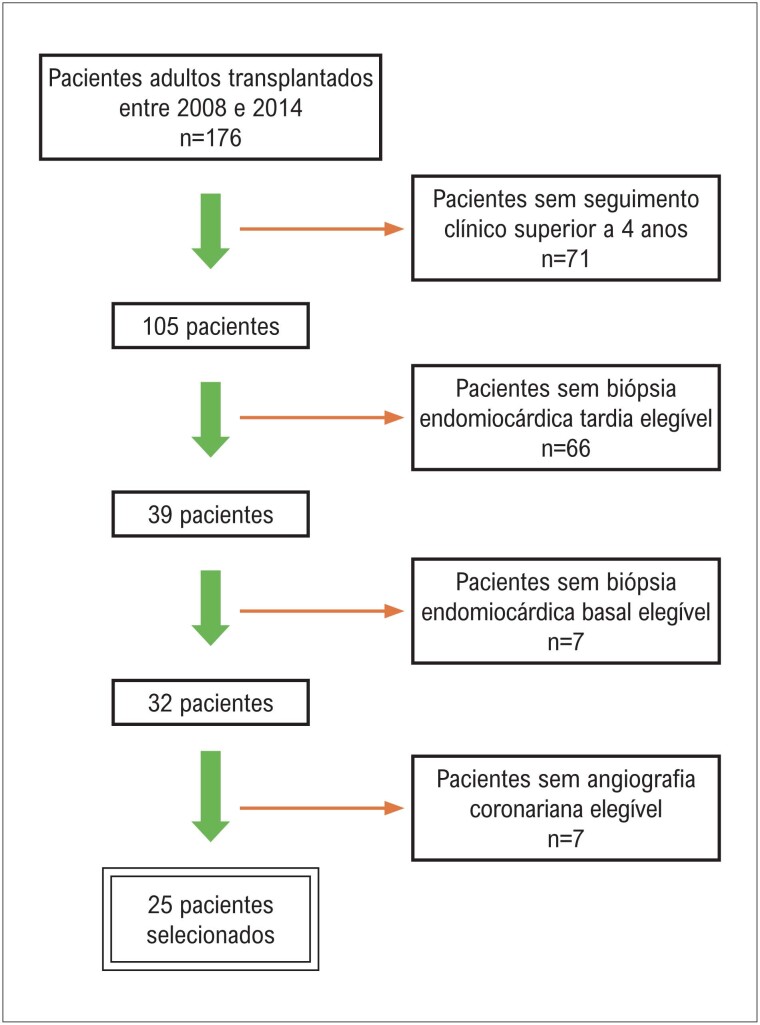
Fluxograma para seleção dos pacientes.

De 2008 a 2014, 176 pacientes adultos foram submetidos a transplante cardíaco em nosso hospital. Os critérios de inclusão para este estudo retrospectivo foram: 1) apresentar seguimento clínico em nosso hospital superior a 4 anos após o transplante; 2) apresentar biópsia endomiocárdica sem rejeição aguda celular significativa (grau ≤ 1R) obtida ao menos 4 anos após o transplante (biópsia tardia), como representativa da condição tardia do coração transplantado; 3) apresentar biópsia endomiocárdica sem rejeição aguda celular significativa (grau ≤ 1R) obtida até o segundo mês após o transplante (biópsia basal), como representativa da condição basal do órgão transplantado (coração normal); e 4) apresentar angiografia coronariana para avaliação de DVE, realizada entre 6 meses antes e 6 meses após a data da coleta da biópsia tardia. Foram selecionados 25/176 (14,2%) pacientes, de acordo com fluxograma representado na Figura 1. Todas as biópsias e angiografias coronarianas foram realizadas no contexto de seguimento clínico rotineiro do paciente transplantado.

A presença e severidade da DVE foi definida de acordo com o consenso de 2010 da Sociedade Internacional de Transplante de Coração e Pulmão (ISHLT),^7^ e classificada como grau 0 (não significativa), grau 1 (leve), grau 2 (moderada) ou grau 3 (grave), a partir de revisão dos laudos e imagens das angiografias.

Cortes histológicos a 3-μm obtidos dos blocos de parafina das biópsias endomiocárdicas foram corados pelo ácido periódico de Schiff (PAS) após digestão com diastase para remoção do glicogênio, com o intuito de ressaltar os grânulos de lipofuscina. A quantidade de lipofuscina no miocárdio foi calculada pela média da área fracionária do citoplasma ocupada pelo pigmento em 4 campos microscópicos não coincidentes, visualizados ao microscópio (Axioskop 2 plus, Zeiss, Jena, Alemanha) com aumento de 1.000 vezes. Utilizou-se o método da contagem de pontos,^9^ mediante superposição aos campos microscópicos de grade computadorizada de 525 pontos equidistantes, com o auxílio do software AxioVision (Zeiss, Jena, Alemanha). A avaliação foi feita por um patologista, sem conhecimento prévio se as biópsias mensuradas eram basais ou tardias.

### Análise estatística

Dados quantitativos foram expressos como média ± desvio padrão ou mediana (percentil 25-percentil 75). Para avaliar a correlação entre idade e quantidade de lipofuscina nas biópsias foi utilizado o teste de correlação de Pearson. O teste de Mann-Whitney foi utilizado para comparação da quantidade de lipofuscina nas biópsias basais e tardias considerando-se a mesma idade cardíaca agrupada por décadas e para comparação da quantidade de lipofuscina das biópsias tardias entre pacientes que apresentavam ou não DVE moderada/grave. Nível de significância foi considerado se p≤0,05.

## Resultados

Quatorze (56%) dos 25 pacientes incluídos neste estudo eram homens. O tempo decorrido desde o transplante até a obtenção da biópsia tardia variou de 4 a 10,1 anos, com mediana de 6,1 (4,43-7,43) anos. A idade cardíaca na época da coleta da biópsia basal (idade do doador), o tempo decorrido do transplante até a coleta da biópsia tardia, a idade cardíaca na época da coleta da biópsia tardia (idade do doador + anos decorridos até a coleta da biópsia tardia), a quantidade de lipofuscina das biópsias basal e tardia e o grau da DVE estão apresentados na Tabela 1.

**Tabela 1:** Idade cardíaca nos tempos basal e tardio do transplante, tempo decorrido até a coleta da biópsia tardia (Δ T), fração de área (%) ocupada pela lipofuscina nas biópsias basal e tardia e grau da DVE dos pacientes

Idade cardíaca basal (anos)	Δ T (anos)	Idade cardíaca tardia (anos)	Lipofuscina basal (%)	Lipofuscina tardia (%)	Grau da DVE
25	6,8	31,8	1,38	3,01	0
38	4,1	42,1	1,88	1,57	3
33	4,0	37,0	0,89	1,76	0
34	6,3	40,3	1,33	1,72	0
29	4,5	33,5	1,32	2,50	0
27	4,9	31,9	0,81	1,53	0
20	4,3	24,3	0,81	1,43	0
15	4,7	19,7	0,67	1,48	0
40	4,1	44,1	1,43	2,62	1
26	4,7	30,7	1,48	1,62	1
37	4,0	41,0	2,03	3,01	0
18	10,8	28,8	0,48	1,24	1
22	9,1	31,1	0,71	2,48	0
43	10,1	53,1	1,36	3,45	2
44	7,6	51,6	2,01	3,82	0
21	6,9	27,9	0,90	2,67	0
43	8,4	51,4	1,87	2,25	2
33	8,2	41,2	1,52	2,05	2
26	5,6	31,6	1,30	2,57	0
19	5,1	24,1	0,34	1,48	0
26	6,7	32,7	1,39	1,26	0
27	4,2	31,2	0,95	1,62	0
23	7,2	30,2	1,00	2,05	3
43	6,9	49,9	2,16	2,34	0
21	6,1	27,1	1,05	2,87	2

DVE: doença vascular do enxerto.

**Figura 2:**
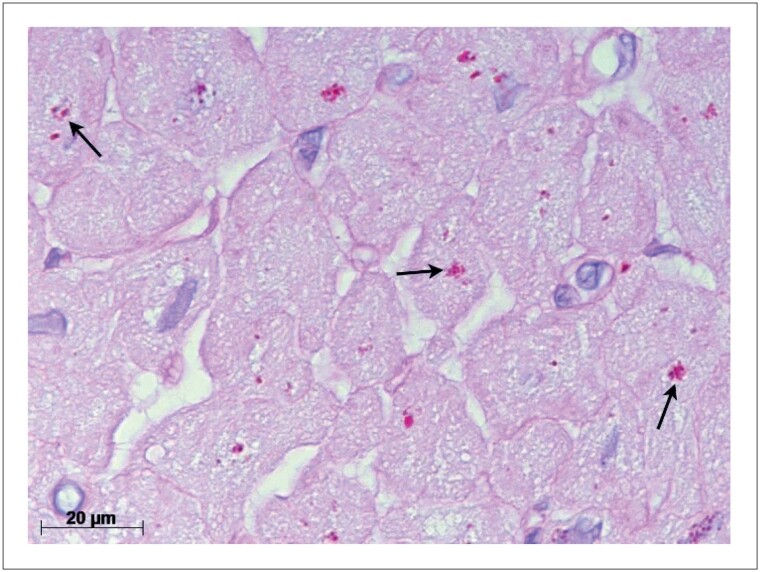
Grânulos de lipofuscina (setas) no citoplasma dos cardiomiócitos corados vermelho-púrpura pelo ácido periódico de Schiff (PAS) após digestão com diastase. Nessa imagem a área fracionária ocupada pela lipofuscina mediu 1,71%.

Imagem representativa de um campo microscópico de biópsia endomiocárdica demonstrando a deposição de lipofuscina no miocárdio (magnificação de 1.000 vezes) está apresentado na Figura 2.

**Figura 3:**
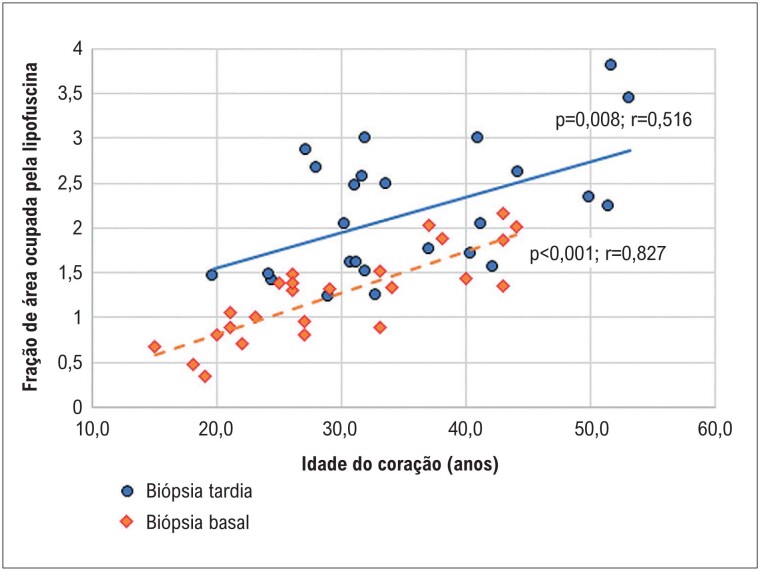
Gráfico de dispersão múltipla relacionando a idade do coração com a fração de área ocupada pela lipofuscina nas biópsias endomiocárdicas basal e tardia.

Houve correlação positiva entre a idade cardíaca e a quantidade de lipofuscina tanto na condição basal (coração normal) como na tardia do coração transplantado. Entretanto, o coeficiente de correlação na condição basal (p<0,001; r=0,827) foi consideravelmente superior ao da condição tardia (p=0,008; r=0,516). Gráfico de dispersão dos dados está apresentado na Figura 3.

A quantidade de lipofuscina nas biópsias tardias foi superior à das biópsias basais considerando-se a mesma idade cardíaca, agrupada por décadas. A mediana da fração de área ocupada pela lipofuscina foi 1,03 (0,83-1,37) nas biópsias basais (n=12) e 1,48 (1,34-2,77) nas biópsias tardias (n=5) dos corações com 20-29 anos de idade; 1,52 (1,11-1,96) nas biópsias basais (n=5) e 1,91 (1,60-2,52) nas biópsias tardias (n=10) dos corações com 30-39 anos de idade; e 1,87 (1,40-2,09) nas biópsias basais (n=5) e 2,20 (1,68-2,72) nas biópsias tardias (n=6) dos corações com 40-49 anos de idade. Entretanto, a diferença da quantidade de lipofuscina entre as biópsias basais e tardias foi estatisticamente significante apenas na década de 20-29 anos de idade cardíaca (p=0,015).

A mediana da fração de área ocupada pela lipofuscina nas biópsias tardias dos pacientes que apresentavam DVE moderada/grave (n=6) ou apresentavam DVE não significativa/leve (n=19) foi 2,15 (1,93-3,02) e 1,76 (1,48-2,62) respectivamente, sem diferença estatística entre os grupos. Pacientes que apresentavam DVE moderada/grave apresentavam idade cardíaca mais elevada (média: 40,8 ± 10,7 anos) que os pacientes que apresentavam DVE não significativa/leve (média: 33,9 ± 8,4 anos), sem diferença estatística entre os grupos.

## Discussão

O pigmento de lipofuscina se origina do dano oxidativo de organelas citoplasmáticas, particularmente mitocôndrias, que são engolfadas pelos lisossomos no processo de autofagia. Alternativamente o pigmento pode se originar diretamente no citoplasma, por proteínas oxidadas não clivadas pelo proteosoma. Como a lipofuscina não se degrada, o pigmento se acumula no citoplasma de células pós-mitóticas de longa sobrevida, refletindo o desgaste celular característico do envelhecimento.^2,3^

Exceto pela caquexia, há controvérsia se a taxa de deposição de lipofuscina no miocárdio pode ser alterada por outros fatores que não o envelhecimento. Em estudo prévio a fração de área ocupada pela lipofuscina no miocárdio não diferiu em pacientes com insuficiência cardíaca de origem isquêmica ou hipertrófica quando comparada a pacientes com coração normal, de mesma idade.^4^ Por outro lado, estudando pacientes jovens com cardiomiopatia dilatada foi descrito que aqueles com melhor fração de ejeção do ventrículo esquerdo apresentaram maiores quantidades de lipofuscina.^10^ Mais recentemente, há relato de acúmulo de lipofuscina nos cardiomiócitos hipertróficos de paciente de 16 anos de idade com defeito de condução cardíaca associada a mutação do gene da subunidade 5 alfa do canal de sódio voltagem-dependente (SCN5A).^11^

Até onde sabemos há um único estudo focando a deposição de lipofuscina no miocárdio após o transplante cardíaco.^8^ Nesse estudo os autores sugeriram que a detecção de lipofuscina em biópsias endomiocárdicas obtidas tardiamente (12 meses) após o transplante cardíaco poderia ser preditiva do desenvolvimento de DVE. Entretanto, a idade dos doadores não foi claramente determinada e a avaliação da lipofuscina nas biópsias foi apenas qualitativa, não quantitativa.

No presente estudo mostramos forte correlação positiva entre a idade e a quantidade de lipofuscina em corações recém transplantados, presumivelmente normais, representados pelas biópsias endomiocárdicas basais. O coeficiente de correlação nessa situação foi 0,827, bastante similar aos coeficientes obtidos em estudos prévios sobre corações normais.^1,4^ Entretanto, o coeficiente de correlação entre a quantidade de lipofuscina e a idade cardíaca foi muito menor (0,516) para as amostras obtidas de corações transplantados há longo tempo (biópsias tardias). Além disso, os depósitos de lipofuscina no miocárdio mostraram-se aumentados após transplante de longa duração quando comparados a corações recém transplantados com a mesma idade, agrupados por década. Esses achados apontam para maior turnover de organelas celulares e maior estresse oxidativo dos cardiomiócitos no transplante de longo tempo de evolução, sugerindo envelhecimento precoce do coração transplantado.

A hipótese de envelhecimento prematuro do coração transplantado, e talvez também de outros órgãos sólidos, é interessante pois poderia ajudar a explicar a relativamente curta sobrevida funcional dos órgãos sólidos transplantados comparada aos órgãos nativos. Entretanto, essa teoria necessita ser confirmada por outros estudos envolvendo maior número de pacientes transplantados, diferentes tipos de transplante, e múltiplos métodos de avaliação do envelhecimento.

Apesar de nosso estudo ser limitado pelo pequeno número de biópsias analisadas e de pacientes que desenvolveram DVE moderada ou grave, não encontramos associação entre a quantidade de lipofuscina no miocárdio dos pacientes transplantados de longa data e o desenvolvimento da DVE.
