# Supplementary material for: Distúrbio Cumulativo da Lipofuscina Miocárdica após Transplante Cardíaco de Longa Evolução: Estudo Baseado em Biópsias Endomiocárdicas
Source: Arq Bras Cardiol. 2023 Mar 27;120(3):e20220313. [Article in Portuguese] doi: 10.36660/abc.20220313 (PMC10263452; doi:10.36660/abc.20220313)
Supplement: Translation

# Translation: Cumulative Disorder of Myocardial Lipofuscin after Long-Term Heart Transplantation: A Study Based on Endomyocardial Biopsies

**Affiliations:** 1 São Paulo SP Brazil Instituto do Coração do Hospital das Clínicas da Faculdade de Medicina da Universidade de São Paulo, São Paulo, SP – Brazil

**Keywords:** Heart transplantation, Lipofuscin, Endomyocardial biopsy

## Introduction

Lipofuscin is a yellowish-brown, non-degradable cytoplasmic pigment composed of highly oxidized proteins, lipids, and metals. Lipofuscin accumulates over time in perennial post-mitotic cells such as cardiomyocytes and is also called “age pigment”.^1-4^

Heart transplantation is an established treatment for patients with severe heart failure refractory to other forms of treatment.^5^ Its major long-term limitation is the development of cardiac allograft vasculopathy (CAV), which is relatively difficult to diagnose and for which few effective treatment options exist.^6,7^ In this context, it is worth mentioning a previous study relating the amount of lipofuscin in the myocardium of heart transplant patients to the development of CAV.^8^

The objective of this study was to evaluate the deposition of lipofuscin in the myocardium after long-term heart transplantation and to investigate the relationship between the amount of lipofuscin in the myocardium and the presence and severity of CAV.

## Methods

From 2008 to 2014, 176 adult patients underwent heart transplantation at our hospital. The inclusion criteria for this retrospective study were: 1) clinical follow-up in our hospital for more than 4 years after transplantation; 2) present endomyocardial biopsy without significant acute cellular rejection (grade ≤ 1R) obtained at least 4 years after transplantation (late biopsy), as representative of the late condition of the transplanted heart; 3) present endomyocardial biopsy without significant acute cellular rejection (grade ≤ 1R) obtained up to the second month after transplantation (baseline biopsy), as representative of the baseline condition of the transplanted organ (normal heart); and 4) present coronary angiography for evaluation of CAV, performed between 6 months before and 6 months after the date of collection of the late biopsy. We selected 25/176 (14.2%) patients, according to the flowchart shown in Figure 1. All biopsies and coronary angiographies were performed in the context of routine clinical follow-up of the transplanted patient.

**Figure 1:** Flowchart for patient selection.

The presence and severity of CAV were defined according to the 2010 consensus of the International Society for Heart and Lung Transplantation (ISHLT),^7^ and classified as grade 0 (not significant), grade 1 (mild), grade 2 (moderate) or grade 3 (severe), based on the review of angiographic reports and images.

Histological 3-*μ*m sections obtained from the paraffin blocks of the endomyocardial biopsies were stained with periodic acid-Schiff (PAS) after digestion with diastase to remove glycogen, to highlight the lipofuscin granules. The amount of lipofuscin in the myocardium was calculated by averaging the fractional area of the cytoplasm occupied by the pigment in 4 non-coincident microscopic fields, visualized under a microscope (Axioskop 2 plus, Zeiss, Jena, Germany) with an X 1,000 magnification. A point counting method was used,^9^ by superimposing a computerized grid of 525 equidistant points to the microscopic fields, with the aid of the AxioVision software (Zeiss, Jena, Germany). A pathologist performed the evaluation without prior knowledge of whether the measured biopsies were baseline or late.

### Statistical analysis

Quantitative data were expressed as mean ± standard deviation or median (25th percentile-75th percentile). Pearson's correlation test was used to evaluate the correlation between age and the amount of lipofuscin in the biopsies. Mann-Whitney rank test was used to compare the amount of lipofuscin in baseline and late biopsies considering the same cardiac age (grouped by decades) and to compare the amount of lipofuscin in late biopsies between patients who had or did not have moderate/severe CAV. The significance level was considered if p≤0.05.

## Results

Fourteen (56%) of the 25 patients included in this study were men. The time elapsed from transplantation to collection of the late biopsy ranged from 4 to 10.1 years, with a median of 6.1 (4.43-7.43) years. Cardiac age at baseline biopsy collection (donor age), the time elapsed from transplantation to late biopsy collection, cardiac age at the time of late biopsy collection (donor age + years elapsed until late biopsy collection), the amount of lipofuscin in the baseline and late biopsies and the grade of CAV are presented in Table 1.

**Table 1:** Cardiac age at baseline and late transplantation period, time elapsed until late biopsy procedure (Δ T), fractional area (%) occupied by lipofuscin in baseline and late biopsies, and CAV grade of patients

Baseline cardiac age (years)	Δ T (years)	Late cardiac age (years)	Baseline biopsy lipofuscin (%)	Late biopsy lipofuscin (%)	CAV grade
25	6.8	31.8	1.38	3.01	0
38	4.1	42.1	1.88	1.57	3
33	4.0	37.0	0.89	1.76	0
34	6.3	40.3	1.33	1.72	0
29	4.5	33.5	1.32	2.50	0
27	4.9	31.9	0.81	1.53	0
20	4.3	24.3	0.81	1.43	0
15	4.7	19.7	0.67	1.48	0
40	4.1	44.1	1.43	2.62	1
26	4.7	30.7	1.48	1.62	1
37	4.0	41.0	2.03	3.01	0
18	10.8	28.8	0.48	1.24	1
22	9.1	31.1	0.71	2.48	0
43	10.1	53.1	1.36	3.45	2
44	7.6	51.6	2.01	3.82	0
21	6.9	27.9	0.90	2.67	0
43	8.4	51.4	1.87	2.25	2
33	8.2	41.2	1.52	2.05	2
26	5.6	31.6	1.30	2.57	0
19	5.1	24.1	0.34	1.48	0
26	6.7	32.7	1.39	1.26	0
27	4.2	31.2	0.95	1.62	0
23	7.2	30.2	1.00	2.05	3
43	6.9	49.9	2.16	2.34	0
21	6.1	27.1	1.05	2.87	2

CAV: cardiac allograft vasculopathy.

A representative image of a microscopic field of an endomyocardial biopsy demonstrating lipofuscin deposition in the myocardium (magnification: X 1,000) is shown in Figure 2.

**Figure 2:** Granules of lipofuscin (arrows) in the cytoplasm of cardiomyocytes stained purple-red by periodic acid-Schiff (PAS) after digestion with diastase. In this image, the fractional area occupied by lipofuscin measured 1.71%.

There was a positive correlation between cardiac age and the amount of lipofuscin in both baseline (normal heart) and late condition of the transplanted heart. However, the correlation coefficient in the baseline condition (p<0.001; r=0.827) was considerably higher than in the late condition (p=0.008; r=0.516). The data scatter plot is shown in Figure 3.

**Figure 3:** Multiple dispersion graph relating the age of the heart with the fractional area occupied by lipofuscin in basal and late endomyocardial biopsies.

The amount of lipofuscin in late biopsies was higher than in baseline biopsies, considering the same cardiac age grouped by decades. The median of the fractional area occupied by lipofuscin was 1.03 (0.83-1.37) in baseline biopsies (n=12) and 1.48 (1.34-2.77) in late biopsies (n=5) of hearts aged 20-29 years; 1.52 (1.11-1.96) in baseline biopsies (n=5) and 1.91 (1.60-2.52) in late biopsies (n=10) of hearts aged 30–39-year; and 1.87 (1.40-2.09) in baseline biopsies (n=5) and 2.20 (1.68-2.72) in late biopsies (n=6) of hearts aged 40-49 years. However, the difference in the amount of lipofuscin between baseline and late biopsies was statistically significant only in the decade of 20-29 years of cardiac age (p=0.015).

The median of the fractional area occupied by lipofuscin in late biopsies of patients with moderate/severe CAV (n=6) or with non-significant/mild CAV (n=19) was 2.15 (1.93-3.02) and 1.76 (1.48-2.62) respectively, with no statistical difference between the groups. Patients with moderate/severe CAV had a higher cardiac age (mean: 40.8 ± 10.7 years) than those with non-significant/mild CAV (mean: 33.9 ± 8.4 years), with no statistical difference between the groups.

## Discussion

The lipofuscin pigment originates from the oxidative damage of cytoplasmic organelles, particularly mitochondria, which are engulfed by lysosomes in the process of autophagy. Alternatively, the pigment may originate directly in the cytoplasm by oxidized proteins not cleaved by the proteasome. Lipofuscin is not degradable, so the pigment accumulates in the cytoplasm of long-lived post-mitotic cells, reflecting the characteristic cellular wear and tear of aging.^2,3^

Except for cachexia, there is controversy as to whether the rate of lipofuscin deposition in the myocardium can be altered by factors other than age. In a previous study, the fractional area occupied by lipofuscin in the myocardium did not differ in patients with heart failure of ischemic or hypertrophic origin when compared to patients with normal hearts of the same age.^4^ On the other hand, studying young patients with dilated cardiomyopathy, it was described that those with better left ventricular ejection fraction had higher amounts of lipofuscin.^10^ More recently, there is a report of lipofuscin accumulation in the hypertrophic cardiomyocytes of a 16-year-old patient with cardiac conduction defect associated with mutation of the sodium voltage-gated channel alpha subunit 5 gene (SCN5A).^11^

To the best of our knowledge, only one study focuses on lipofuscin deposition in the myocardium after heart transplantation.^8^ In that study, the authors suggested that the detection of lipofuscin in endomyocardial biopsies obtained late (12 months) after heart transplantation could be predictive of the development of CAV. However, the age of the donors was not clearly determined, and the evaluation of lipofuscin in the biopsies was only qualitative, not quantitative.

In the present study, we showed a strong positive correlation between age and the amount of lipofuscin in newly transplanted hearts, presumably normal, represented by baseline endomyocardial biopsies. The correlation coefficient in this situation was 0.827, quite similar to the coefficients obtained in previous studies on normal hearts.^1,4^ However, the correlation coefficient between the amount of lipofuscin and cardiac age was much lower (0.516) for the samples obtained from hearts transplanted a long time ago (late biopsies). In addition, lipofuscin deposits in the myocardium were shown to be increased after long-term transplantation when compared to newly transplanted hearts of the same age, grouped by decade. These findings point to a higher turnover of cell organelles and greater oxidative stress of cardiomyocytes in long-term transplants, suggesting early aging of the transplanted heart.

The hypothesis of premature aging of the transplanted heart, and perhaps also of other solid organs, is interesting as it could help to explain the relatively short functional survival of transplanted solid organs compared to native organs. However, this theory needs to be confirmed by other studies involving a greater number of transplanted patients, different types of transplants, and multiple methods of assessing aging.

Although our study was limited by the small number of biopsies analyzed and the number of patients who developed moderate or severe CAV, we found no association between the amount of lipofuscin in the myocardium of long-term transplant patients and the development of CAV.
